# Supplementary material for: A CNN-RNN Framework for Crop Yield Prediction
Source: Front Plant Sci. 2020 Jan 24;10:1750. doi: 10.3389/fpls.2019.01750 (PMC6993602; doi:10.3389/fpls.2019.01750)
Supplement: Supplementary file 1 [file DataSheet_1.pdf]

# Supplementary Material

## 1 SUPPLEMENTARY TABLES AND FIGURES

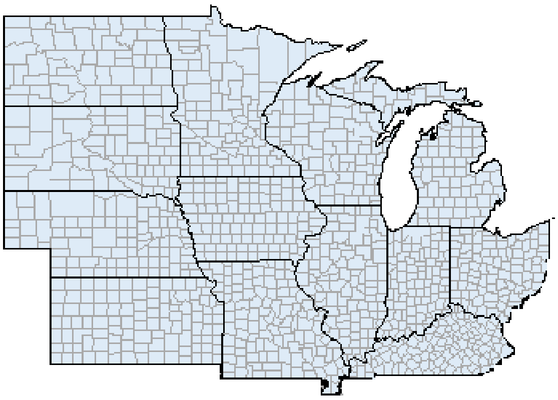

Figure S1: Map of the Corn Belt in the United States.

| Summary statistics | Corn | Soybean |
|---|---|---|
| Total number of locations | 1,176 | 1,115 |
| Year range | 1980-2018 | 1980-2018 |
| Mean yield | 120.71 | 38.22 |
| Standard deviation of yield | 38.13 | 10.47 |
| First quartile of yield | 94.3 | 31.0 |
| Median yield | 121.0 | 38.0 |
| Third quartile of yield | 147.0 | 45.5 |
| Minimum yield | 11.0 | 6.0 |
| Maximum yield | 246.7 | 82.3 |
| Number of weather components | 6 | 6 |
| Number of soil components | 14 | 14 |
| Number of management components | 1 | 1 |
| Number of observations | 41,070 | 36,277 |

Table S1: Summary statistics of the data over all years. The unit of yield is bushels per acre

| W-CNN structure | | | | |
|---|---|---|---|---|
| Input size | 52 × 1 | | | |
| Layer name | FS | NF | S | P |
| Conv1 | 9 | 8 | 1 | valid |
| Average pooling 1 | 2 | - | 2 | valid |
| Conv2 | 3 | 12 | 1 | valid |
| Average pooling 2 | 2 | - | 2 | valid |
| Conv3 | 3 | 16 | 1 | valid |
| Average pooling 2 | 2 | - | 2 | valid |
| Conv3 | 3 | 20 | 1 | valid |
| Average pooling 2 | 2 | - | 2 | valid |
| Output size | 20 × 1 | | | |

| S-CNN structure | | | | |
|---|---|---|---|---|
| Input size | 10 × 1 | | | |
| Layer name | FS | NF | S | P |
| Conv1 | 3 | 4 | 1 | valid |
| Average pooling 1 | 2 | - | 2 | valid |
| Conv2 | 3 | 8 | 1 | valid |
| Average pooling 2 | 2 | - | 2 | valid |
| Conv3 | 2 | 12 | 1 | valid |
| Output size | 12 × 1 | | | |

Table S2: The left and the right tables show the detailed stucture of the W-CNN and S-CNN, respectively. FS, NF, S, and P stand for filter size, number of filter, stride, and padding, respectively.

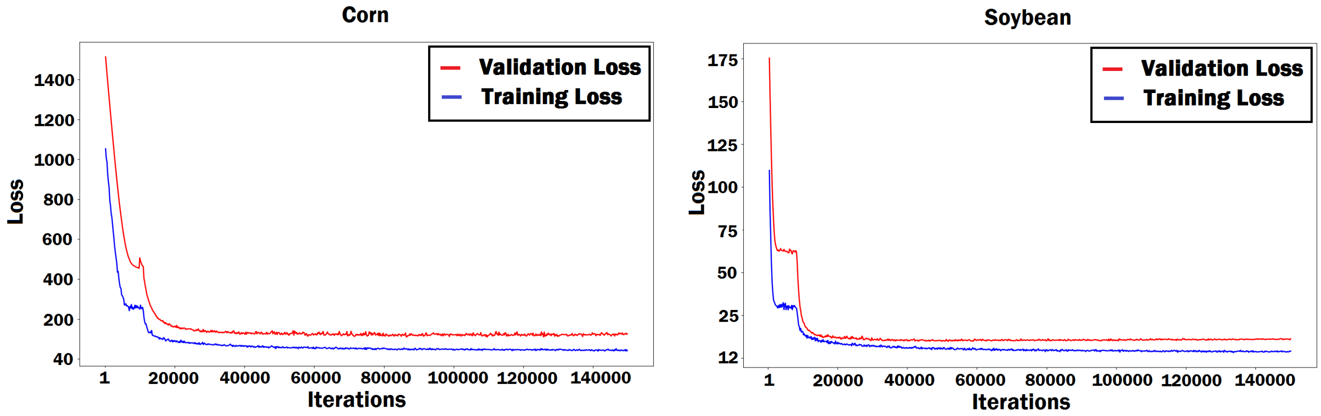


Figure S2: The left and right plots show the plots of the training loss and validation loss for corn and soybean yield predictions of the year 2018, respectively. We used Huber loss which combines both squared loss and absolute loss.

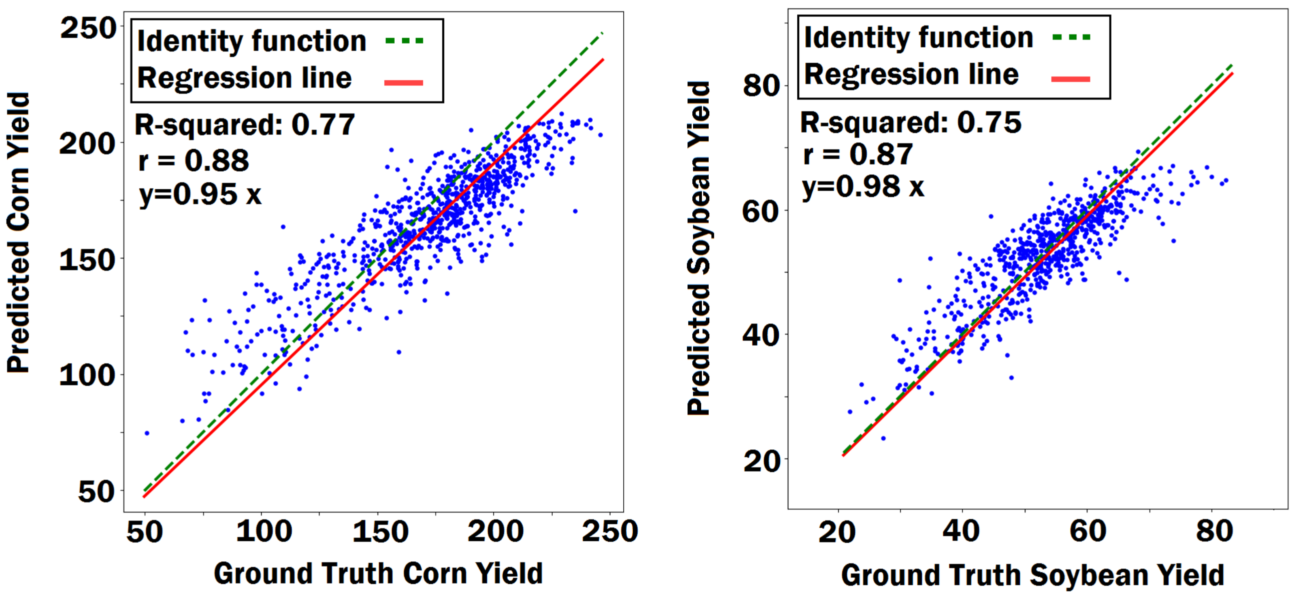


Figure S3: The left and right plots show the plots of the ground truth yield versus the predicted yield for corn and soybean yield predictions of the year 2018, respectively. The unit of yield is bushels per acre.

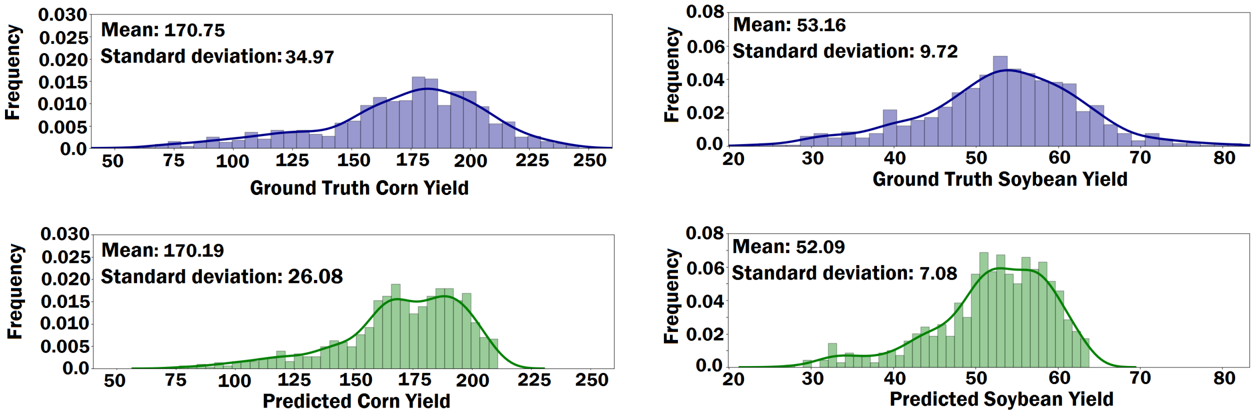


Figure S4: The left and right plots show the probability density functions of the ground truth yield and the predicted yield for corn and soybean yield predictions of the year 2018, respectively. The plots indicate that CNN-RNN model can approximately preserve some of the distributional properties of the ground truth yield. The unit of yield is bushels per acre.

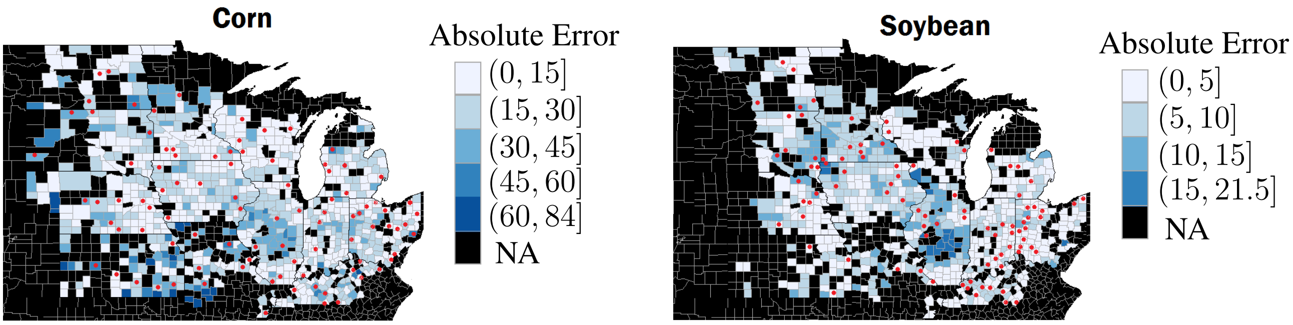


Figure S5: The left and right maps show the absolute prediction errors of the year 2018 for corn and soybean yield predictions, respectively. Counties with red dots indicate the untested counties. The counties in black color inside the cornbelt indicate that ground truth yields were not available for these counties for the year 2018. The unit of error is bushels per acre.
